# Care complexity, perceptions of complexity and preferences for interprofessional collaboration: an analysis of relationships and social networks in paediatrics

**DOI:** 10.1186/s12909-024-05304-6

**Published:** 2024-03-25

**Authors:** Lisa-Maria van Klaveren, Vincent Geukers, Rien de Vos

**Affiliations:** 1 Institute for Education and Training, Amsterdam Universities Medical Centres location University of Amsterdam, Meibergdreef 15, 1105 AZ Amsterdam, The Netherlands; 2 Emma Children’s Hospital, Amsterdam Universities Medical Centres location University of Amsterdam, Meibergdreef 15, 1105 AZ Amsterdam, The Netherlands

**Keywords:** Interprofessional collaboration, Complexity, Social network analysis, Interprofessional education

## Abstract

**Background:**

In the context of increasingly intricate healthcare systems, professionals are compelled to collaborate within dynamically changing interprofessional teams. Moreover, they must adapt these collaborative processes to effectively and efficiently manage the evolving complexity of care needs. It remains unclear how professionals determine care complexity and relate this complexity to their preferences for interprofessional collaboration (IPC). This study investigated the relationships between care complexity, professionals’ perceived complexity and IPC preferences, and examined the variation in individual and team characteristics of IPC-practices across different levels of complexity in paediatric care.

**Methods:**

In an online questionnaire, 123 healthcare professionals working at an academic tertiary children’s hospital scored their perceptions of complexity and preferences for IPC. They also selected family and various professions as members of the interprofessional (IP-) team based on thirteen patient cases. We employed conjoint analysis to systematically model the complexity of case descriptions across the five domains of the International Classification of Functioning, Disability and Health (ICF). Additionally, we applied social network analysis to identify important professions, crucial connectors and influential professions in the IP-team, and to describe the cohesiveness of IP-teams.

**Results:**

Modelled case complexity, professionals’ perceived complexity and IPC preferences were positively associated. We found large inter-individual variations in the degree of these associations. Social network analysis revealed that the importance and influence of professions was more equally distributed when case complexity increased. Depending on the context and complexity of the case, different professions (e.g. medical doctors, social professionals, extramural professionals) were considered to be more crucial connectors within the IP-team. Furthermore, team cohesion was positively associated with modelled and perceived care complexity.

**Conclusions:**

In conclusion, our study contributes to the existing knowledge by integrating task-specific insights and broadening the use of conjoint and social network analysis in the context of IPC. The findings substantiate the contingency theory that relates characteristics of IPC to care complexity, offering quantified insights into how IP-teams adapt to situational needs. This understanding of relationships and variations within IPC holds crucial implications for designing targeted interventions in both clinical and health profession education contexts. Consequently, it contributes to advancements in healthcare systems.

**Supplementary Information:**

The online version contains supplementary material available at 10.1186/s12909-024-05304-6.

## Background

In recent decades, healthcare systems have undergone substantial transformations, giving rise to complexities that surpass the knowledge and skills of single health professions [1, 2]. As a result of advancements in modern medicine, conditions that were once lethal have transformed into chronic illnesses. This transformation has led to increased survival rates and aging of the population, with higher prevalence and severity of chronic and comorbid conditions, also among paediatric patients [3, 4]. A growing number of children and adolescents with chronic conditions face the risk of cognitive, social, and emotional setbacks. This compromises their educational paths and places strain on family finances. Consequently, these children and adolescents may encounter fewer opportunities in life compared to their peers without chronic conditions [5]. As a consequence, traditional *multi*ple mono-disciplinary consultations without cohesive coordination can result in unclear decision-making and leadership ambiguity. This may give rise to conflicts in advices, counter-effective therapies, and unsafe transitions between health institutions, adding to communicative and logistic burdens of patients and their families.

Interprofessional collaboration (IPC) is proposed as a promising approach for navigating the growing complexities of healthcare effectively and efficiently [6, 7]. It involves the collaborative creation and implementation of an integrated care plan, engaging patients, their families, doctors from various medical specialties, nurses as well as allied health and social professionals within and across diverse care settings [8, 9]. To facilitate the integration of diverse perspectives and expertise in IPC, Glader and colleagues [10] advocate for the adoption of the International Classification of Functioning, Disability and Health (ICF) [11] as standard framework for care planning and evaluation in complex paediatric care. The ICF can be a useful tool for describing various health and welfare domains, encompassing both personal and environmental factors and their influence on patients’ functioning [12]. By providing a more holistic biopsychosocial model and, thus, a shared language, Snyman et al. [13] argue that the ICF facilitates interprofessional (IP-) teams, engaging patients and their families, in constructing a shared understanding of complex care needs. This understanding can subsequently be incorporated into and executed as a comprehensive care plan.

IPC is not a fixed entity but varies across different types of practice. Reeves et al. [14] argue that the degree of interprofessional collaborative (IPC-) practice is positively related to healthcare complexity. They suggest adopting a contingency approach as a theoretical foundation to more effectively delineate the variations in IPC. Specifically, they classify IPC practices into four levels, ranging from networks as the least intense form of IPC to coordination, collaboration, and teamwork as the most comprehensive and intensive practices. Differences between practices can be explained by six elements: shared team identity, clear roles and goals, interdependence, integration, shared responsibility and team tasks [15]. In unpredictable complex care, all elements are crucial and very close interprofessional teamwork is necessary to deliver appropriate care. When complex care becomes more predictable, shared identity, integration and interdependence become less important during IPC-practices such as collaboration and coordination. In predictable and low complex care, loosely connected IP-teams in networks are sufficient [15].

Thus, to deliver appropriate care that effectively and efficiently aligns priorities and circumstances [16], professionals and IP-teams need to attune their IPC-practices to changing situations considering team constellations and roles, and the degrees of integration and interdependence between their members. To ensure high levels of team functioning and performance within and across patients’ changing care needs, these complex adaptive IP-teams need to know when and how to deploy members’ expertise and skills to match situational needs [17].

In practice, however, perceptions of complexity of care and preferences for IPC seem to be more permeable. Professional, social, physical and task-related gaps persist in integrating expertise into joint care planning [18]. Also, challenges on various levels, including ideological, organizational, structural or relational aspects hinder the realization of IPC’s full potential [19]. These unsolved issues may not only result in compromised quality of care and care outcomes for patients and their families [20], but may also lead to reduced job satisfaction among health and social professionals [21]. A comprehensive understanding of professionals’ perceptions of complexity and related preferences can enhance IPC, thus contributing to advancements of healthcare systems. This improvement may lead to enhanced care experiences, cost reductions, increased professional satisfaction and improved health equity [22].

The goal of this study is twofold: First, to expand the understanding of the relationships between case complexity, professionals’ perceived case complexity and IPC preference in practice. Second, to gain insights into the variations in individual and team characteristics of IPC-practices across levels of case complexity.

With this study, we aim to contribute to the existing literature in three ways: First, we empirically test the theoretical contingency approach to IPC [15]. Second, we add task specific insights to existing knowledge from questionnaires on professionals’ general attitudes to IPC [23]. Third, we extend recent applications of social network analysis to IPC-practices to substantiate the conceptualization of appropriate care as an effort of complex adaptive IP-teams [17, 24, 25].

## Methods

In line with the two goals mentioned above, we formulated three research questions: (1) What are the associations between modelled case complexity, professionals’ perceived case complexity and IPC preference in terms of professionals’ inclination towards integrated care planning and their indicated need of an IP-team meeting? (2) What is variation in individual characteristics of IPC-practices in terms of important professions, crucial connectors and influential professions between levels of modelled case complexity? (3) What are the associations between (modelled and perceived) case complexity and team characteristics of IPC-practices in terms of cohesion?

### Design

To answer these research questions, we conducted an observational study at a tertiary, academic paediatric hospital. Participants were administered a web-based questionnaire through Qualtrics (Provo, UT), comprising thirteen systematically modelled patient case descriptions with varying complexities. These cases were presented in a random order to the participants. For each case, participants answered three required and two follow-up questions. After finishing the last patient case, participants answered a short series of demographic questions about their professional background and number of years of experience.

The study was pre-registered prior to accessing the data on the Open Science Framework (https://osf.io/6cr83). Data was collected between December 2022 and June 2023.

### Participants and setting

Participants were professionals working in direct patient care at the Emma Children’s Hospital that is situated in the Amsterdam Universities Medical Centres, the Netherlands. The Emma Children’s Hospital houses a spectrum of specialized fields. Specifically within the realm of urology, the Emma Children’s Hospital exhibits distinctive proficiency in managing congenital disorders affecting the kidneys, bladder and urethra. We included medical doctors, nursing professionals, other health professionals (e.g. dieticians or physiotherapists), and social professionals (e.g., paediatric psychologists or social workers) (ISO) [26]. From all professions, we also included professionals-in-training (e.g., registered nurses in training for paediatric nurse, registered doctors in training for paediatrician). Participants were recruited through email invitations. To encourage responses from those who had not yet participated, two reminder emails were sent at four and eight weeks following the initial invitation. No incentives were offered for participation.

### Case descriptions

The case descriptions were systematically modelled using elements of conjoint analysis [27]. This scientific method is considered an optimal approach for measuring the value that professionals place on features of products or situations in economics and in healthcare [28, 29]. Set in the context of paediatric urology and the management of congenital disorders in particular, we modelled case descriptions based on five domains of the ICF for Children and Youth [30]: (1) personal factors, (2) environmental factors, (3) body functions and anatomical structures, (4) activities, and (5) participation. Conform to the ICF-framework, we chose to group body functions and structures together, and to separate activities and participation. Within each domain, we chose two representative elements of the ICF-coding system with three levels of complexity (low, medium, complex). The selection of elements within the ICF-domains as well as the operationalization into concrete examples was done by the research team consisting of a psychologist (LK), paediatrician-intensivist (VG) and clinical epidemiologist (RV). During this iterative process of feedback and adaptation, the validity and applicability of the preliminary case descriptions were frequently verified with the input of two paediatric nurses, a paediatrician, a paediatric urologist and a physiotherapist. In a pilot involving two specialized nurses, two nurses in training and two medical doctors in training, we evaluated the comprehensibility of case descriptions, questions and response options. Subsequently, adjustments were made based on the feedback received, and the materials were finalized.

As the ICF does not specify *Personal factors* [31–33], we followed Heerkens et al. [34] and operationalized them as personal characteristics in terms of 1a) chronic health condition and 1b) medical needs. Following the ICF, *Environmental factors* were operationalized as facilitators or barriers to functioning in terms of 2a) social security and 2b) social network. *Body functions and anatomical structures* were operationalized as impairments in body functioning in terms of 3a) metabolic or urogenital functions and 3b) sensory or mental functions. *Activities* were operationalized as limitations in activities in terms of 4a) mobility and 4b) self-care. *Participation* was operationalized as restrictions in participation in terms of 5a) education and 5b) leisure activities. Table 1 shows the operationalization of the five ICF-domains, featuring two representative elements each across three levels of complexity. The complete list of verbatim translated cases is available in the supplementary material.

#### Modelled case complexity

To describe the modelled complexity, we calculated the sum and product score per case based on the three complexity levels (*low* = 1 to *high* = 3) for each of the five ICF-domains. This led to potential ranges of sum scores, spanning from 5 to 15, and product scores, ranging from 1 to 243.

#### Case selection

The combination of five ICF-domains with three levels of complexity yielded a total of 243 potential case descriptions. Considering respondent feasibility and employing conjoint analysis methodology, thirteen cases were randomly chosen for the final questionnaire, controlling for the complexity weight of individual ICF-domains. In each ICF-domain, the lowest and highest complexity levels were incorporated five times in the selected cases. Medium levels were featured three times, except in the ICF-domain *Activities*. In this domain, the medium level was included five times, while the high level occurred three times.

**Table 1:** Modelled complexity of five ICF-domains with three levels of complexity

Complex	Personal factors	Environ. factors	Body functions	Activities	Participation
low	no chronic condition	parents with a good social network	urinates spontaneously	mobilizes without support	engages in team sport
	no medication	average income	headache	sufficient oral intake	attends regular education
medium	asthma	single parent with unstable social network	white blood cells in urine, fever	mobilizes with support	engages in solitary hobby
	home medication	welfare benefit	pain when urinating	insufficient oral intake	attends special education
high	hypoventilation syndrome	divorced parents without social network	hydronephrosis	does not mobilize	no sport or hobby engagement
	night-time non-invasive ventilation	statutory debt restructuring	confused, pain in left flank	no oral intake	high school absenteeism

### Outcomes

To address the first research question, we measured professionals’ perceived case complexity and IPC preference in terms of their inclination towards integrated care planning and their indicated need of an IP-team meeting.

#### Perceived case complexity

To measure professionals’ perceived care complexity, we employed the following question for each case: “How do you evaluate the situation of the described patient at this moment?” Responses were scored on a continuous scale from zero (very simple) to 100 (very complex).

#### IPC preference

We differentiated in professionals’ IPC preference using two measures to gain a more nuanced understanding: (1) their inclination towards integrated care planning and (2) their indicated need for an IP-team meeting. First, to measure professionals’ inclination towards integrated care planning, we utilized the following question for each case: “How do you evaluate the importance of the creation of an integrated care plan for the described patient?” Responses were scored on a continuous scale from zero (very unimportant) to 100 (very important). An integrated care plan was defined as a shared care plan that includes all healthcare domains and integrates perspectives of involved team members [35]. Second, to measure professionals’ need for an IP-team meeting, we posed the following question for each case: “Do you think that a multi- or interdisciplinary round-table consultation should take place to create an integrated care plan for the described patient?” Participants selected one of the following options: “No, go to the next patient case” (*score* = 1) or “Yes, choose team members for this consultation” (*score* = 2). Importantly, we chose the “multi- or interdisciplinary round-table consultations” as an indicator for IPC due to its widespread usage and recognition within the local context of our study [36]. By adopting this locally established terminology, we aimed to enhance precision and clarity in the questionnaire.

To address the second and third research question, we operationalized the characteristics of IPC-practices. Individual characteristics were measured based on important professions, crucial connectors and influential professions, while team characteristics were assessed by team cohesion.

#### Characteristics of IPC-practice

To measure the characteristics of IPC-practice, we employed the following question for each case: “A multi- or interdisciplinary round-table consultation will take place to create an integrated care plan for the described patient. Who do you think should certainly be present at this consultation?” Participants chose professions as team members that should certainly or optional be present. To enhance clarity and convenience, options were divided into five groups: family, nursing, medical, other and external. The first group included one option: parents. In line with the principles of IPC, we intentionally included the option for parents as potential team members participating in multi- or interdisciplinary round-table consultations, reflecting the widely accepted view of the parents’ role in paediatrics. The nursing category refers to nursing professionals and encompassed three options: paediatric nurse, specialist nurse urology and other nurse specialist (open text box). The medical category pertained to medical doctors and also included three options: paediatrician, paediatric urologist and other medical specialist (open text box). The other group encompasses other health and social professionals and comprised the following options: dietician, physiotherapist, social worker, pedagogical care provider, psychologist and other health professional (open text box). The external or extramural group refers to professionals outside the paediatric hospital and included two options: general practitioner and other external health professional (open text box). This question was only presented to participants for cases in which they had indicated the need multi- or interdisciplinary round-table consultation.

Utilizing social network analysis [37], we created social networks for each case based on the selection of team members deemed to be essential by participants. The social networks are depicted with node sizes and edge weights. Node sizes indicate the proportion of participants who selected a specific IP-team member as certainly present during the multi- or interdisciplinary round-table consultation. Meanwhile, edge weights signify the number of participants who indicated that the two connected nodes should both be certainly present IP-team members.

To quantify the individual characteristics of IPC-practice, we calculated (1) degree centrality, (2) betweenness centrality and (3) closeness centrality for all potential IP-team members. First, to find important professions, we calculated the degree centrality for each member for each case by counting the number of edges or co-occurrences with other IP-members for each individual IP-team member [37]. The metric provides insights into the overall structure of an IP-team by revealing which professions are more central in terms of connectivity. Second, to identify crucial connectors that coordinate the information flow in the IP-team, we assessed betweenness centrality by calculating the proportion of all shortest paths in the IP-team that pass through a given member [37]. The metric is valuable for detecting critical professions that mediate interactions within an IP-team. Third, to find influential professions, we calculated closeness centrality by assigning a score to each member based on its sum of shortest paths between this member and all other colleagues in the IP-team [37]. The metric is particularly useful for identifying professions that are strategically positioned to facilitate quick communication within a complex system, and it contributes valuable insights into the overall efficiency and accessibility of the IP-team.

To quantify the team characteristics of IPC-practice, we calculated network density by dividing the actual number of connection times two by all potential interactions [37]. This metric characterizes the degree of cohesion or fragmentation within the network, offering valuable insights into the potential for group formation and information diffusion. The verbatim translated questionnaire is available in the supplementary material.

### Ethical considerations

The study was approved by the Institutional Review Board of the Amsterdam Universities Medical Centres, location Amsterdam Medical Centre, who waived the need for a full ethical review (W22_295#22.356). Participants were informed about the study and asked for consent before answering the digital questionnaire. Participants were informed about the study during staff meetings, in newsletters and through email. Informed consent was given immediately before answering the questionnaire.

### Analysis

The data underwent two pre-processing steps. First, to account for individual participants’ general perceptions and preferences, mean-centred (MC) values were computed for each respondent concerning perceived complexity and inclination towards integrated care planning [38]. Second, to address non-normal distributions, rank transformations were applied to all variables when appropriate [39].

To describe the sociodemographic properties of the sample, we computed frequencies for professional background and training status, along with measures of central tendency for work experience.

To assess the associations between modelled case complexity, professionals’ perceived case complexity and IPC preference in terms of their inclination towards integrated care planning and their indicated need of an IP-team meeting, we calculated Pearson product-moment correlation coefficients. These coefficients were computed between rank-transformed (RT) sum and product scores of modelled complexity, MC/RT scores of perceived complexity, MC/RT scores of inclination towards integrated care planning, and the scores of indicated need for an IP-team meeting. To account for the repeated measures design, correlations were calculated per participant. Weighted means for correlation coefficients and p-values were derived across all participants, adjusting for the number of completed cases [40]. Weighted confidence intervals were employed to describe inter-individual variations [41]. To depict inter-individual variation, we generated scatterplots with smoothing lines using local regression (LOESS) for continuous scores [42] and generalized linear models (GLM) for binary scores [43]. Following the contingency approach proposed by Reeves et al. [14], we predicted that RT sum and product scores of modelled complexity, MC/RT scores of perceived complexity, MC/RT scores of inclination towards integrated care planning, and the RT scores of indicated need for an IP-team meeting were positively correlated.

To determine the variation in individual characteristics of IPC-practices in terms of important professions, crucial connectors and influential professions between levels of modelled case complexity,, we compared the degree, betweenness and closeness centrality per IP-team member between five networks. The networks were chosen based on the (1) minimum, (2) lower quartile, (3) median, (4) upper quartile and (5) maximum scores of modelled complexity. We visualized individual characteristics of IPC-practice for these networks using the Kamada-Kawai drawing algorithm for undirected graphs [44]. Taking the perspective of adaptive teams [17], we expected that the degree, betweenness and closeness centrality of IP-team members differed across levels of case complexity.

Lastly, to assess the associations between (modelled and perceived) case complexity and team characteristics of IPC-practices in terms of cohesion, we calculated Kendall’s rank correlations coefficients. These coefficients were computed for sum and product scores of modelled complexity, averaged MC perceived complexity and network density. Following the perspective of care teams as adaptive systems [17], we predicted that sum and product scores of modelled complexity, averaged MC scores of perceived complexity, network density were positively correlated with network density.

Statistical significance was set at the level of *p* <.05. All analyses were performed using R (4.0.3, 2020-10-10).

## Results

### Population

In total, 439 professionals (197 nursing professionals, 174 medical doctors, and 68 health or social professionals) working at the Emma Children’s Hospital were invited to participate in this observational study, of whom 123 (28%) consented to participate. We included 110 participants (52 nursing professionals, 46 medical doctors, and 12 health or social professionals) who completed the questionnaire for more than 50% of the cases in the final analyses. Twenty-eight participants were in specialist training. The median professional experience was 8 years (*IQR* = 15).

### Associations between modelled case complexity, perceived case complexity and IPC preferences

Descriptive analysis revealed that MC scores of perceived complexity ranged between − 58.31 and 42.77 across all cases. Overall MC scores of inclinations towards integrated care planning varied between − 69.39 and 50.77. In total, participants opted for an (IP-team meeting 732 times (52.51%), while there were 662 occurrences (47.49%) when no IP-team meeting was indicated. Table 2 shows the descriptive statistics for each of the thirteen cases.

**Table 2:** Descriptive statistics for modelled case complexity, perceived case complexity and IPC preferences by case

Selected case	Modelled Complexity		MC^*^ perceived Complexity	IPC preferences	
				MC^*^ inclination integrated care planning	Indicated need IP-team meeting
No.^#^	Sum Median (IQR)	Product Median (IQR)	Mean (SD)	Mean (SD)	% yes
82	6	2	-19.29 (13.04)	-17.85 (14.20)	11.11
64	8	6	-13.69 (12.41)	-10.12 (14.43)	45.71
34	8	6	-5.96 (14.05)	-9.93 (14.40)	21.70
21	9	9	-1.38 (12.15)	-2.62 (15.80)	45.79
17	9	12	-3.54 (13.46)	-8.49 (14.70)	35.51
175	9	12	-3.22 (10.66)	-3.53 (12.91)	44.76
218	10	18	-8.65 (14.62)	-4.44 (15.77)	48.15
60	10	18	2.18 (11.57)	1.87 (14.33)	49.07
171	11	27	9.75 (10.63)	7.88 (13.52)	65.09
201	11	36	6.47 (11.95)	6.61 (14.26)	65.74
131	11	48	5.52 (9.51)	6.47 (12.31)	71.30
241	13	81	13.09 (10.80)	14.29 (13.29)	85.19
153	13	108	17.92 (11.14)	18.99 (12.37)	91.82
	10 (2)	18 (27)	-0.06 (10.64)	-0.07 (10.59)	52.38 (23.15)

^#^ No. refers to the number of the randomly selected case from 243 possible cases.

^*^ MC refers to the mean-centred scores.

Associations with modelled case complexity.

RT scores of modelled case complexity and MC/RT scores of perceived complexity were significantly correlated in terms of sum score (*R*_*weighted*_ = 0.639, 95% CI_weighted_ [0.250, 1], *p*_*weighted*_ = 0.027) and product score (*R*_*weighted*_ = 0.630, 95% CI_weighted_ [0.240, 1], *p*_*weighted*_ = 0.033). RT scores of modelled case complexity were also significantly correlated with MC/RT scores of inclinations towards integrated care planning (sum score: *R*_*weighted*_ = 0.621, 95% CI_weighted_ [0.225, 1], *p*_*weighted*_ = 0.035; product score: *R*_*weighted*_ = 0.618, 95% CI_weighted_ [0.223, 1], *p*_*weighted*_ = 0.040). The 95% weighted confidence interval showed inter-individual variations, and indicated that, on population level, the correlation coefficients between RT scores of modelled complexity and the two outcome measures ranged from 0.250/0.240 to 0.225/0.223 and 1. Post-hoc analyses revealed no correlation between individuals’ coefficients and their variation in MC/RT scores, as measured by standard deviations. RT scores of modelled case complexity and scores of indicated IP-team meeting were not significantly correlated (sum score: *R*_*weighted*_ = 0.505, 95% CI_weighted_ [0.061, 1], *p*_*weighted*_ = 0.076; product score: *R*_*weighted*_ = 0.503, 95% CI_weighted_ [0.058, 1], *p*_*weighted*_ = 0.078).

**Fig. 1:**
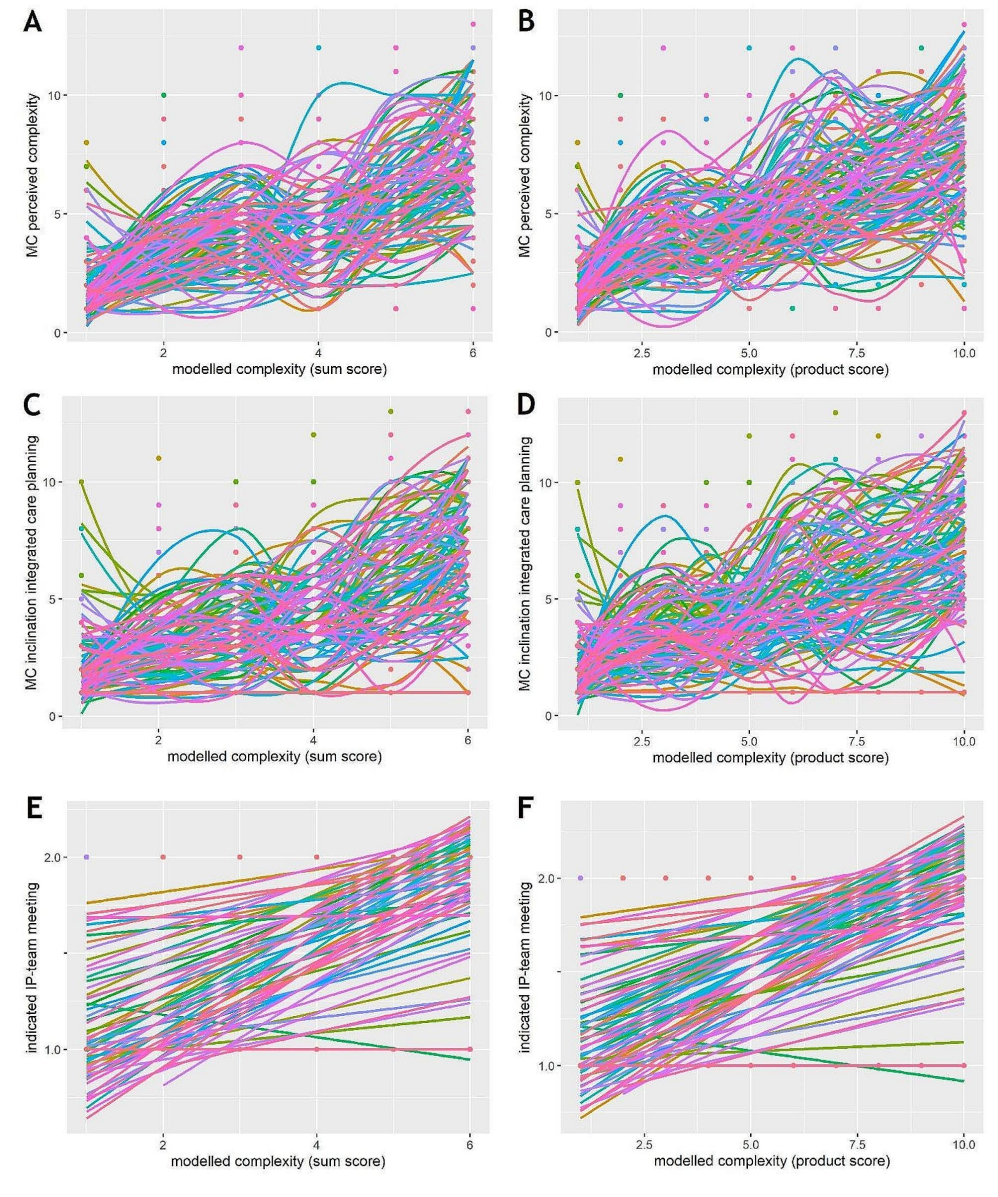
Scatterplots for modelled case complexity, perceived case complexity and IPC preferences with smoothing lines per participant Note: The scatterplots show the RT scores of all variables. Labels of the x- and y-axes refer to the range of possible ranks within a variable

While Fig. 1 illustrates general trends, indicating that higher RT sum and product scores of modelled case complexity correspond to higher MC/RT scores of perceived complexity (Fig. 1A-B), higher MC/RT scores of inclination towards integrated care planning (Fig. 1C-D), and higher RT scores of indicated need for an IP-team meeting (figure E-F), individual trajectories appear to be diverse. This inter-individual variation in MC/RT scores of perceived complexity and inclination towards integrated care planning is particularly evident towards high RT modelled complexity. Conversely, the opposite trend is observed for the RT score of need for an IP-team meeting. In order to explore the visual trends, and inter individual variations, we compared the interquartile ranges across modelled complexity ranks. Smaller interquartile ranges (*IQR* = 1–2.5) were observed for MC/RT scores of perceived complexity at lower modelled complexity (ranks two, three, and four, Fig. 1A-B). Wider interquartile ranges (*IQR* = 4) were noted at rank one as well as at high ranks. A similar analysis was carried out for MC/RT scores of inclination towards integrated care planning. High interquartile ranges (*IQR* = 4–5) were only observed at the highest ranks of modelled complexity, while the lowest interquartile ranges (*IQR* = 2) were again seen at ranks three and four (Fig. 1C-D). This supports the visual trend that the inter-individual variation increases with increased complexity.

Associations between perceived case complexity and IPC preferences.

MC/RT scores of inclination towards integrated care planning were correlated with MC/RT scores of perceived complexity (*R*_*weighted*_ = 0.779, 95% CI [0.510, 1], *p*_*weighted*_ = 0.009), as well as with scores of indicated need for IP-team meetings (*R*_*weighted*_ = 0.679, 95% CI [0.230, 1], *p*_*weighted*_ = 0.032). The correlation between MC/RT scores of perceived complexity and RT scores of indicated need for IP-team meeting was not significant (*R*_*weighted*_ = 0.565, 95% CI [0.147, 1], *p*_*weighted*_ = 0.056). Post-hoc analyses revealed no correlation between individuals’ coefficients and their variation in MC/RT scores, as measured by standard deviations.

**Fig. 2:**
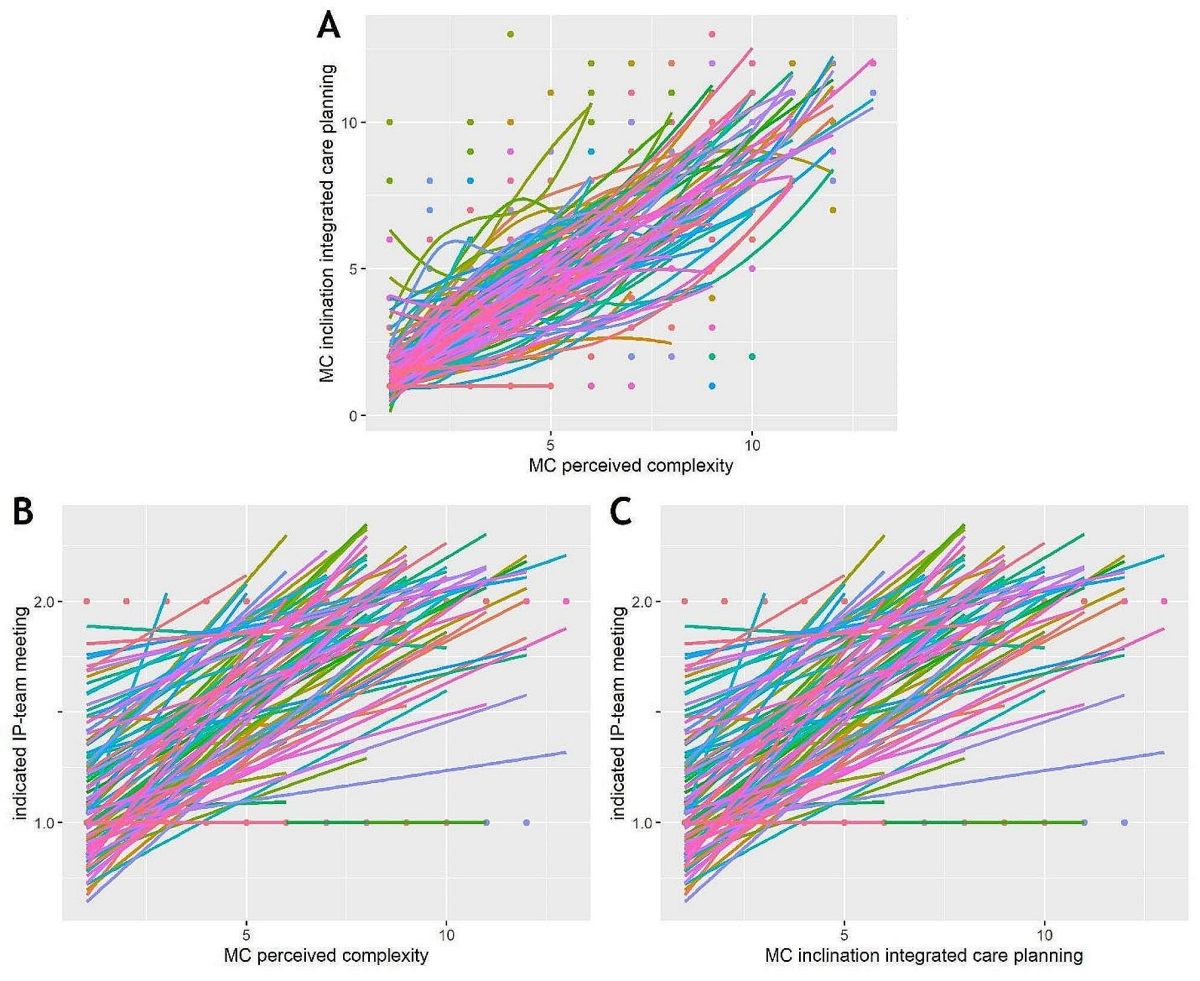
Scatterplots for perceptions of complexity and preference for IPC with smoothing lines per participant Note: The scatterplots show the RT scores of all variables. Labels of the x- and y-axes refer to the range of possible ranks within a variable

Figure 2A-C illustrates the inter-individual variations in the associations between MC/RT perceived complexity, MC/RT scores of inclination towards integrated care planning and RT scores of indicated need for IP-team meeting. While some participants appear to have strong general tendency towards IP-team meetings in general, independent from care complexity, others indicate the need for a team meeting only on few occasions.

### Variation of individual characteristics of IPC-practice between levels of modelled case complexity

**Fig. 3:**
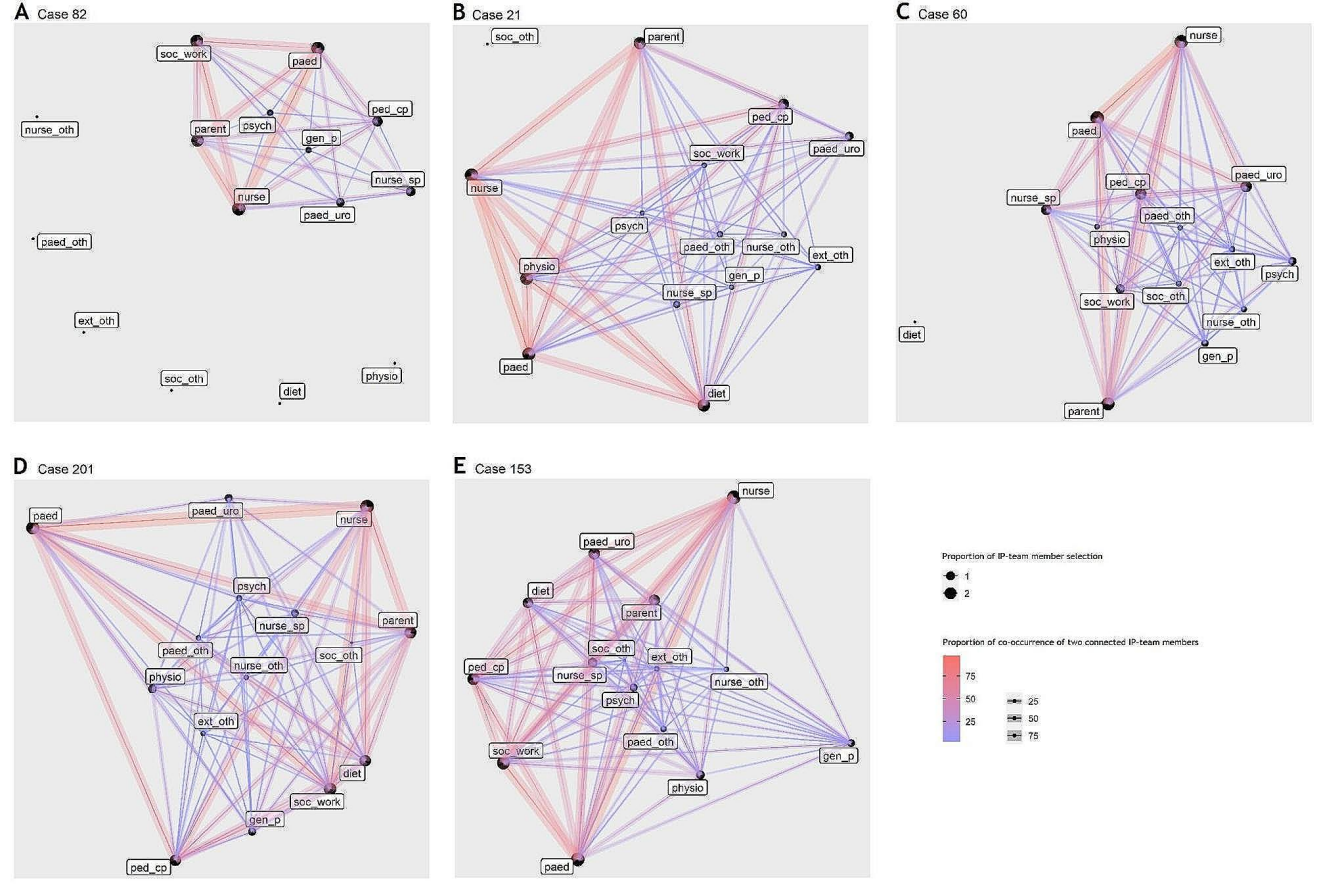
Social networks for five cases based on modelled complexity (min, Q1, median, Q3, maximum)

Degree centrality of IP-team members varied between cases of different complexity levels. While all selected IP-team members were highly connected and equally important in IPC-practices for maximum complex cases (case 153, *range*_*DC*_ = 11–14), less members were involved in IPC-practices with more differences in their individual importance between them at the lowest modelled complexity (case 82, *range*_*DC*_ = 0–8). Betweenness centrality also seemed to differ between cases. For instance, while the psychologist appeared to be a crucial connector (*BC*_*82*_ = 11.000, *BC*_*21*_ *=* 26.583) in cases 82 and 21, the paediatric urologist (*BC* = 19.357) and nurse specialist (*BC* = 24.968) were most influential in the network of case 201. Moreover, other professionals such as medical and nursing specialists from other discipline such as pulmonology, psychiatry or nephrology, and extramural professionals such as school officials, youth services and home care representatives seemed to be more crucial connectors when complexity increased. Lastly, there seemed to be a decreasing trend in average closeness centrality related to rising complexity. Figure 3 visualizes the IPC-practices for the five exemplary cases (3 A minimum, 3B first quartile, 3 C median, 3D second quartile, 3E maximum). Table 3 provides descriptive statistics for the centrality measures.

**Table 3:** Degree, betweenness and closeness centrality for each potential IP-team member per case

	Case 82			Case 21			Case 60			Case 201			Case 153		
IP-team member	DC	BC	CC	DC	BC	CC	DC	BC	CC	DC	BC	CC	DC	BC	CC
Parent	8	0.500	0.071	13	0.000	0.022	13	0.000	0.020	14	0.000	0.021	14	0.000	0.014
Paed.^#^ nurse	8	0.500	0.071	13	0.000	0.021	13	0.000	0.019	14	0.000	0.023	14	0.000	0.009
Nurse spec.^*^ urology	7	0.500	0.071	11	0.000	0.029	13	0.000	0.026	13	19.357	0.033	14	0.000	0.017
Other paed. nurse spec.	0	0.000	NA	9	1.000	0.032	11	1.250	0.030	13	24.968	0.036	13	0.000	0.014
Paediatrician	8	0.500	0.071	13	0.000	0.022	13	0.000	0.023	13	0.000	0.015	14	0.000	0.009
Paed. urologist	7	1.000	0.077	10	0.000	0.024	13	0.450	0.028	12	0.000	0.025	14	0.000	0.012
Other medical specialist	0	0.000	NA	13	15.750	0.038	11	14.433	0.037	13	7.855	0.031	14	10.000	0.017
Dietician	0	0.000	NA	13	0.000	0.023	0	0.000	NA	14	0.833	0.024	14	0.000	0.012
Physiotherapist	0	0.000	NA	13	0.000	0.023	7	13.950	0.033	13	0.000	0.025	13	0.000	0.012
Social worker	7	0.000	0.059	10	0.750	0.033	13	4.200	0.031	14	1.675	0.026	14	0.000	0.010
Medical pedagogical care provider	8	1.750	0.083	13	0.000	0.024	13	3.100	0.031	13	0.000	0.024	14	0.000	0.011
Psychologist	6	11.000	0.100	10	26.583	0.040	11	0.000	0.026	13	9.891	0.034	14	2.583	0.019
Other health professional	0	0.000	NA	0	0.000	NA	9	14.767	0.036	5	22.865	0.029	11	73.42	0.022
General practitioner	5	6.000	0.083	10	8.833	0.037	12	0.833	0.029	13	0.000	0.024	13	0.000	0.008
Other external professional	0	0.000	NA	9	0.583	0.03	12	19.317	0.037	11	29.594	0.034	14	17.42	0.020
median (IQR)	6 (7.5)	0.5 000 (0.75)	0.071 (0.012)	11 (3)	0.000 (0.875)	0.027 (0.001)	12 (2)	0.833 (9.075)	0.030 (0.007)	13 (0.5)	0.833 (14.62)	0.024 (0.008)	14 (0.5)	0.000 (1.29)	0.012 (0.007)

^#^ paediatric

^*^ specialist

### Associations between (modelled and perceived) case complexity and team characteristics of IPC-practice

Modelled case complexity, both in terms of sum and product scores, was positively correlated with network density (sum score: *tau* = 0.641, *p* =.004; product score: *tau* = 0.641, *p* =.003). Additionally, perceived case complexity was positively correlated with network density (*tau* = 0.560, *p* =.004). This finding suggests that the degree of IPC-practice increased with rising complexity.

## Discussion

The goal of this study was to expand the understanding of the relationship between case complexity, professionals’ perceptions of complexity, and preferences for IPC, and to gain insights into the variations in individual and team characteristics of IPC-practices across levels of case complexity. Building on the contingency approach outlined by Reeves et al. [14], our results, indicating positive associations between modelled case complexity, perceived case complexity and IPC preference, lend support to this theory. Nevertheless, the observed variance between individuals’ associations could not be entirely accounted for. For instance, individual variation in perceptions and IPC preferences across cases was not related to individual associations. Consequently, we hypothesize that one or more additional pertinent factors might be associated with professionals’ perceptions and preferences for IPC [45]. Potential factors that may explain the observed inter-individual variation include professional background and work experience, proficiency in interprofessional competencies as well as personal believes and motivations. Moreover, the extent of inter-individual variation differed across levels of modelled complexity. This might also indicate that separate ICF-domains influence individual perceptions of complexity and preferences for IPC differently.

Taking the perspective of complex adaptive teams [17], our data substantiates the theory by revealing that individual characteristics of IPC-practices vary across case complexity. Social network analyses revealed that important professions, crucial connectors and influential professions seemed to change when care became more complex. First, the importance of members from various professions became more equally distributed among the whole IP-team. In correspondence with the contingency theory, this might be an indicator for an increase in interdependence and shared responsibility between members that is essential in close IPC-practices in order to effectively deal with high degrees of complexity [14, 15]. Second, the profession of crucial connectors changed based on care situations. This apparent adaption to situational needs might signal the clarity of roles, goals and tasks with IP-teams that is necessary in all IPC-practices [15]. Third, the influence of members from various professions became more equally distributed with increasing complexity. This might afford quick communication between all team members in order to reach optimal efficiency and accessibility to interprofessional expertise. In line with the contingency theory, this equal distribution may suggest an increased need for integration that is necessary to provide appropriate care in highly complex situations [15]. Overall, team cohesions was positively associated with modelled and perceived complexity. This might indicate that group formation or shared team identity becomes stronger when complexity increases. To conclude, social network analysis might provide empirical indices for the four levels of IPC-practice described in the contingency theory [14].

### Strengths and limitations

The findings of this study should be interpreted in light of several limitations. Firstly, the use of paper-based cases, while designed as a practical approximation of professionals’ intentions in real-life scenarios and carefully modelled, may limit the generalizability of the results to their actual actions in real-world settings. However, the research question can hardly be systematically answered by other means, and the used technique of case descriptions and conjoint analysis is a valid way of elucidating preferences [29, 46]. Additionally, the range of case complexity in this study is somewhat restricted by selected elements with the five ICF-domains and their complexity levels. While there are alternative approaches to model health complexity, we chose to utilize the ICF model as it has been proposed as a comprehensive framework for complex care [10, 11]. Although a broader array of professionals could be considered for the study population, we believe that the professions included in this study are representative of complex paediatric care in our setting. Besides, IPC has been a longstanding and common practice in paediatrics. Therefore, the study of perceptions of complexity and preferences for IPC is extremely relevant within this healthcare domain, that is generally believed ‘doing well’ in this respect. Although the results cannot be transferred to adult medicine unequivocally, they can be of great relevance to those fields that are still at the brink of developing IPC communities.

To our knowledge this is one of the few study underpinning theories behind IPC. Such underpinning is crucially important since information on intended actions provide evidence for large efforts and costly efforts made in healthcare to improve health outcomes in view of complexity. This study particularly supports the contingency theory of Reeves et al. [14, 15] and theories of complex adaptive teams [17]. It provides advanced methodological and statistical techniques to quantify IPC-practice. Additionally, the findings suggest substantial inter-individual variations, and thus complex responses, as well as show the complexity of IP-teams. This information bears significant implications to advance IPC in clinical and (continuous) health profession education contexts.

### Implications for interprofessional collaboration and education

To optimize IPC in clinical contexts, these insights may serve as a starting point for the development of effective network models customized to address continuously changing care needs for the delivery of appropriate care. Grounded in clinical outcomes, these models can act as valuable tools for making informed decisions on structuring IPC processes effectively and efficiently over time [46, 47]. Specifically, these models may offer insights into decisions related to team compositions and processes by identifying crucial team members and determining the appropriate degrees of IPC-practices. This information may also provide relevant guidance for policy makers and healthcare leaders in adjusting policies and regulations to create healthcare environments that promote IPC.

To foster IPC in (continuous) health profession educational contexts, interventions should be designed to align perceptions of complexity and preferences for ICP and to develop a shared terminology among (future) professionals within and across various healthcare professions, patients and their families [36, 47, 48]. Especially, interprofessional education (IPE) may have the potential to enhance (future) professionals’ comprehension of care complexity as degrees of interrelatedness [49] and IP-teams as complex adaptive systems [17, 50, 51]. This may help (future) professionals and teams develop necessary interprofessional competencies, and to effectively and efficiently adapt to continuously changing complexities [14, 16, 52].

Future directions.

To further validate the theory of Reeves et al. [15], future studies using similar methodology could be expanded towards the field of adult complex care, rehabilitation and community care. To examine inter-individual variation, mixed method approaches could prove useful to investigate determinants such as sociodemographic data, professional and personal value systems, and interprofessional competencies. To substantiate the relevance of the contingency approach for appropriate care, future studies could also examine associations between complexity, IPC-practices and health outcomes. Our social network research was carried out from the perspective of complex adaptive IP-teams [17], such research could be further expanded by longitudinal and interventional studies following real-life actions of IP-teams over longer periods of time to better understand (the development of) team adaptiveness and performance in changing circumstances. These enhancements will contribute to a more comprehensive understanding of relationships and variations IPC processes. This understanding can advance healthcare systems by enhancing care experiences, reducing costs, fostering professional satisfaction and increasing health equity [22].

## Conclusion

This study provides empirical support for the contingency theory underlying IPC processes and their determinants. It offers quantified insights into the ways in which complex adaptive IP-teams may attune to situational needs in the context of care complexity. Through systematic examination utilizing conjoint analysis and expanding recent applications of social network analysis IPC, we contribute to a more comprehensive understanding of care complexity and IPC-practices.

### Electronic supplementary material

Below is the link to the electronic supplementary material.

Supplementary Material 1

Supplementary Material 2

## Data Availability

All data generated or analysed during this study will be made publicly available on the Open Science Framework (https://osf.io/6cr83).

## Abbreviations

BC: betweenness centrality
CC: closeness centrality
DC: degree centrality
ICF: International Classification of Functioning, Disability and Health
IP: Interprofessional
IPC: Interprofessional collaboration
IQR: Interquartile range
MC: Mean-centred
RT: Rank-transformed
SD: Standard deviation
