# Supplementary material for: Care complexity, perceptions of complexity and preferences for interprofessional collaboration: an analysis of relationships and social networks in paediatrics
Source: BMC Med Educ. 2024 Mar 25;24:334. doi: 10.1186/s12909-024-05304-6 (PMC10962203; doi:10.1186/s12909-024-05304-6)
Supplement: Supplementary file 1 — Supplementary Material 1 [file 12909_2024_5304_MOESM1_ESM.pdf]

A. Modelled cases descriptions

Context of the case (same for all cases):
Patient (6 years), pyeloplasty for unilateral ureteropelvic junction (UPJ) obstruction (left) 3 days ago, procedure was uncomplicated. Double J stent (left) placed, urinary catheter removed this morning. The patient is known in the Emma Children's Hospital. Points of attention during the bedside round:

Individual case descriptions:

<u>Case 17</u>

Medical history	asthma with home medication
Body functions	hydronephrosis, pain in left flank, confused
ADL	mobilizes with support, insufficient oral intake
General	attends mainstream education, engages in team sport
Family	parents with good social network, average income

<u>Case 21</u>

Medical history	hypoventilation syndrome, night-time non-invasive ventilation
Body functions	urinates spontaneously, headache
ADL	does not mobilize, no oral intake
General	attends mainstream education, engages in team sport
Family	parents with good social network, average income

<u>Case 34</u>

Medical history	no chronic condition, no medication
Body functions	hydronephrosis, pain in left flank, confused
ADL	mobilizes with without support, sufficient oral intake
General	attends special education, engages in solitary hobby
Family	parents with good social network, average income

<u>Case 60</u>

Medical history	hypoventilation syndrome, night-time non-invasive ventilation
Body functions	white blood cells in urine, pain when urinating, fever
ADL	mobilizes with without support, sufficient oral intake
General	high school absenteeism, no sport or hobby engagement
Family	parents with good social network, average income

<u>Case 64</u>

Medical history	no chronic condition, no medication

Body functions	urinates spontaneously, headache
ADL	mobilizes with support, insufficient oral intake
General	high school absenteeism, no sport or hobby engagement
Family	parents with good social network, average income

Case 82

Medical history	no chronic condition, no medication
Body functions	urinates spontaneously, headache
ADL	mobilizes with without support, sufficient oral intake
General	attends mainstream education, engages in team sport
Family	single parent with unstable social network, welfare benefit

Case 131

Medical history	asthma with home medication
Body functions	white blood cells in urine, pain when urinating, fever
ADL	does not mobilize, no oral intake
General	attends special education, engages in solitary hobby
Family	single parent with unstable social network, welfare benefit

Case 153

Medical history	hypoventilation syndrome, night-time non-invasive ventilation
Body functions	hydronephrosis, pain in left flank, confused
ADL	mobilizes with support, insufficient oral intake
General	high school absenteeism, no sport or hobby engagement
Family	single parent with unstable social network, welfare benefit

Case 171

Medical history	hypoventilation syndrome, night-time non-invasive ventilation
Body functions	hydronephrosis, pain in left flank, confused
ADL	mobilizes with without support, sufficient oral intake
General	attends mainstream education, engages in team sport
Family	divorced parent without social network, statutory debt restructuring

Case 175

Medical history	no chronic condition, no medication
Body functions	white blood cells in urine, pain when urinating, fever
ADL	mobilizes with support, insufficient oral intake
General	attends mainstream education, engages in team sport
Family	divorced parent without social network, statutory debt restructuring

Case 201

Medical history	hypoventilation syndrome, night-time non-invasive ventilation
Body functions	urinates spontaneously, headache
ADL	mobilizes with support, insufficient oral intake
General	attends special education, engages in solitary hobby
Family	divorced parent without social network, statutory debt restructuring

Case 218

Medical history	asthma with home medication
Body functions	urinates spontaneously, headache
ADL	mobilizes with without support, sufficient oral intake
General	high school absenteeism, no sport or hobby engagement
Family	divorced parent without social network, statutory debt restructuring

Case 241

Medical history	no chronic condition, no medication
Body functions	hydronephrosis, pain in left flank, confused
ADL	does not mobilize, no oral intake
General	high school absenteeism, no sport or hobby engagement
Family	divorced parent without social network, statutory debt restructuring
