# Supplementary material for: Care complexity, perceptions of complexity and preferences for interprofessional collaboration: an analysis of relationships and social networks in paediatrics
Source: BMC Med Educ. 2024 Mar 25;24:334. doi: 10.1186/s12909-024-05304-6 (PMC10962203; doi:10.1186/s12909-024-05304-6)
Supplement: Supplementary file 2 — Supplementary Material 2 [file 12909_2024_5304_MOESM2_ESM.pdf]

Emma Kinderziekenhuis
Amsterdam UMC

Example case:

Patient (6 years), pyeloplasty fpr unilateral ureteropelvic junction (UPJ) obstruction 3 days ago, procedure was uncomplication. Double J stent (left) placed, urinary catheter removed this morning. Patient is known in the Emma Children's Hospital. Points of attention during morning bedside round:

**Medical history**	astma with home medication
**Body functions**	white blood cells in urine, pain when urinating, fever
**ADL**	mobilzes with support, oral intake insufficient
**General**	attends speciaal education, engages in solitary hobby
**Family**	single parent with unstable social network, welfare benefit

How do you evaluate **the situation of the described patiënt** at this moment?

Use the slider to indicate your response from very simple (0) to very complex (100).

very simple 0 | 10 | 20 | 30 | 40 | neither simple, nor complex 50 | 60 | 70 | 80 | 90 | very complex 100

How do you evaluate the importance **of creating an <u>integrated careplan</u> for the described patient**?

Use the slider to indicate your response from very unimportant (0) to very important (100).

very unimportant 0 | 10 | 20 | 30 | 40 | neither unimportant, nor important 50 | 60 | 70 | 80 | 90 | very important 100

Do you think that **a multi- or interdisciplinary round-table consultation** should take place **to create an <u>integrated careplan</u> for the described patient**?

By selecting the "No" button, you indicate that <u>no</u> multi- or interdisciplinary round-table consultation is needed. You then proceed to the next case. By selecting the "Yes" button, you indicate that a multi- or interdisciplinary round-table consultations is required for the described patient. You will then proceed to the next page to select the team members who should be present at this consultation

NO<br>*Go to the next case*	YES<br>*Select team members for this consultation*

A multi- or interdisciplinary round-table consultation will take place **to create an <u>integrated care plan</u>**. Who do you think should certainly be present during this consultation?

By selecting the "optional" or "certainly" button, you can indicate whether a person should "optional" or "certainly" present during the consultation to create an integrated care plan. If you don't think that a person need to attend the consultation, you can leave the button set to "not".

	not	optional	certainly
**FAMILY**			
Parent(s)	○	○	●
**NURSING**			
Paediatric nurse	○	○	●
Nurse specialist urology	○	●	○
Nurse specialist, other:	●	○	○
**MEDICAL**			
Paediatrician	○	○	●
Paediatric urologist	●	○	○
Medical specialist, other:	●	○	○
**OTHER**			
Dietician	●	○	○
Physiotherapist	○	○	●
Social worker	○	●	○
Pedagogical care provider	●	○	○
Psychologist	○	○	●
Other healthcare professional:	●	○	○
**EXTERNAL**			
General practitioner	○	○	●
External healthcare professional: School official	○	●	○
	not	optional	certainly
